# Paraventricular thalamus‐insular cortex circuit mediates colorectal visceral pain induced by neonatal colonic inflammation in mice

**DOI:** 10.1111/cns.14534

**Published:** 2023-11-23

**Authors:** Fu‐Chao Zhang, Ying‐Xue Wei, Rui‐Xia Weng, Qi‐Ya Xu, Rui Li, Yang Yu, Guang‐Yin Xu

**Affiliations:** ^1^ Jiangsu Key Laboratory of Neuropsychiatric Diseases and Institute of Neuroscience Soochow University Suzhou Jiangsu P. R. China; ^2^ Department of Gastroenterology The First Affiliated Hospital of Soochow University Suzhou Jiangsu P. R. China; ^3^ Department of Anesthesiology The First Affiliated Hospital of Soochow University Suzhou Jiangsu P. R. China

**Keywords:** glutamatergic neurons, insular cortex, irritable bowel syndrome, neural circuit, paraventricular thalamus, visceral pain

## Abstract

**Aims:**

Irritable bowel syndrome (IBS) is a common functional gastrointestinal disorder, but its pathogenesis remains incompletely understood, particularly the involvements of central nervous system sensitization in colorectal visceral pain. Our study was to investigate whether the paraventricular thalamus (PVT) projected to the insular cortex (IC) to regulate colorectal visceral pain in neonatal colonic inflammation (NCI) mice and underlying mechanisms.

**Methods:**

We applied optogenetic, chemogenetic, or pharmacological approaches to manipulate the glutamatergic^PVT‐IC^ pathway. Fiber photometry was used to assess neuronal activity. Electromyography activities in response to colorectal distension (CRD) were measured to evaluate the colorectal visceral pain.

**Results:**

NCI enhanced c‐Fos expression and calcium activity upon CRD in the IC^Glu^, and optogenetic manipulation of them altered colorectal visceral pain responses accordingly. Viral tracing indicated that the PVT^Glu^ projected to the IC^Glu^. Optogenetic manipulation of PVT^Glu^ changed colorectal visceral pain responses. Furthermore, selective optogenetic modulation of PVT projections in the IC influenced colorectal visceral pain, which was reversed by chemogenetic manipulation of downstream IC^Glu^.

**Conclusions:**

This study identified a novel PVT‐IC neural circuit playing a critical role in colorectal visceral pain in a mouse model of IBS.

## Graphical abstract

A direct PVT‐IC glutamate neural circuit playing a critical role in visceral hypersensitivity in a mouse model of IBS. This work will strengthen fundamental research on the involvement of central nervous system in visceral hyperalgesia and provide potential therapeutic targets toward effective treatment.

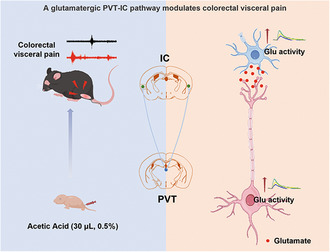

## 1 INTRODUCTION

The prevalence of chronic colorectal visceral pain in clinical practice is as high as 10%–25%.^1^ Irritable bowel syndrome (IBS) is a common functional gastrointestinal disorder characterized by visceral pain, abdominal bloating, and abnormal bowel movements.^2^, ^3^ The incidence of IBS has been increasing in recent years,^4^ whereas the etiology of IBS is not completely understood, which prevents effective treatments and thus makes a negative impact on the physiological and psychological well‐being of affected individuals^5^, ^6^ and increases the burden of the health care system. Currently, most research on chronic colorectal visceral pain focuses on the peripheral aspects, with limited research addressing the contribution of the central nervous system.^7^, ^8^, ^9^, ^10^, ^11^ Therefore, exploring the regulatory mechanisms of the central nervous system on IBS colorectal visceral pain may promote understanding of IBS pathogenesis and identifying new therapeutic targets.

Colorectal visceral pain signals arised from visceral nociceptors with their cell bodies in the dorsal root ganglia (DRG) are transmitted to the spinal dorsal horn. After processing in the spinal dorsal horn, the pain signals are transmitted to the periphery and cognitive centers through multiple pathways.^12^, ^13^ Our previous study suggests that the insular cortex (IC) is involved in the formation of chronic visceral pain in a rat model of IBS.^14^ However, the upstream nuclei that transmit visceral pain signals to the IC are unknown. Among the upstream nuclei projecting to the IC, particular attention should be given to the paraventricular thalamus (PVT), as it serves as an important relay station for ascending peripheral signals.^15^ PVT is involved in the regulation of animal wakefulness^16^, ^17^ and development of colorectal visceral pain induced by substance P in acute pancreatitis.^18^

As such, we hypothesized that glutamatergic neurons in the PVT‐IC circuit might be involved in the formation of colorectal visceral pain in IBS. This study employed in vivo fiber optic recording, optogenetics, chemogenetics, and viral tracing techniques to investigate whether the PVT‐IC circuit was involved in regulating colorectal visceral pain in neonatal colonic inflammation (NCI) mice and underlying mechanisms.

## 2 MATERIALS AND METHODS

### 2.1 Experimental animals

All experimental animals used in this study were male and female SPF‐grade C57BL/6J mice. Wild‐type mice were purchased from Beijing Vital River Laboratory Animal Technology Co., Ltd., while VGluT2‐ires‐Cre knock‐in (C57BL/6J), and transgenic mice were purchased from Shanghai Model Organisms Center, Inc. Female mice were only used for breeding purposes, due to potential analgesic effects of estrogen.^19^, ^20^ Male mice used in the experiments were aged 6–12 weeks with a weight range of 20–26 g. All experiments were approved by the Institutional Animal Care and Use Committee of Soochow University. The care and handling of all mice followed the guidelines of the International Association for the Study of Pain.

### 2.2 Neonatal colonic inflammation (NCI)

Mice aged 7–9 weeks were mated in a ratio of 1 male to 3 females per cage until obvious abdominal swelling was observed in the female mice, at which point the pregnant females were housed individually.^21^ NCI modeling was performed on postnatal day 10. A homemade rectal injector was used to inject 0.5% acetic acid (prepared using physiological saline) at a distance of 1 cm from the anus into the colorectum, with each mouse receiving an injection of 30 μL. After the acetic acid injection, the injector was left in the colorectum for 8–10 s. The experiments were conducted when the offspring reached 6 weeks of age and met the weight requirements.^22^ The control group (CON group) received an equivalent volume of medical‐grade saline injected into the colorectum.

### 2.3 Colorectal visceral pain threshold testing

The colorectal visceral pain threshold in mice was determined based on abdominal withdrawal reflex in response to colorectal distension (CRD) as described previously.^23^, ^24^ In brief, the mice were lightly anesthetized with isoflurane to allow a homemade inflatable balloon (1.5 cm in length) coated with vaseline to be gently inserted into the colorectum with the end of the balloon 0.5 cm from the anus. The catheter of the balloon was securely attached to the base of the mouse's tail. The mice were then placed in individual clear acrylic restrainers for acclimation. The balloon catheter was connected to a sphygmomanometer, and the balloon was slowly inflated until a slight contraction of the mouse's abdomen was observed. This procedure was repeated five times with 5‐min intervals, and the reading on the sphygmomanometer was recorded and averaged as the colorectal visceral pain threshold of the mice. The colorectal visceral pain threshold testing was performed on the same NCI and control mice tested from week 6 to 14. The tests were conducted in a blinded manner.

### 2.4 Immunofluorescence

Prior to the formal experiment, the mice were placed in an individual transparent box to acclimate to the environment for 1 h. After acclimation, the mice were subjected to CRD stimulation at 60 mmHg. CRD stimulation was applied every 3 min for a duration of 20 s, repeated 5 times. After the stimulation, the mice were allowed to recover for 1 h and then deeply anesthetized for perfusion. The perfusion solution consisted of 0.9% physiological saline and 4% paraformaldehyde. The brains of the mice were sliced into 25 μm thick sections using a cryostat following sucrose dehydration.

Immunofluorescence staining was performed as follows: the brain sections were washed with PBS three times (10 min each time). A blocking solution containing 7% donkey serum, 0.3% Triton X‐100, and 0.05% sodium azide was applied for 1 h at room temperature. The sections were then incubated with primary antibodies overnight at 4°C. After incubation, the brain sections were washed with PBS three times (10 min each time), followed by incubation with secondary antibodies for 1 h at room temperature. The brain sections were washed with PBS three times (10 min each time), and finally, the sections were mounted with DAPI‐containing mounting medium (ZH1021, VECTASHIELD). The antibodies used were as follows: Anti‐c‐Fos (sc‐271243, Santa Cruz Biotechnology), Anti‐Glutamate (G6642, Sigma), Anti‐GABA (A2062, Sigma), Anti‐GFP (ab6662, Santa Cruz Biotechnology), ALEXA FLUORTM488 Donkey Anti‐Rabbit IgG (H + L) (2376850, Thermo Fisher Scientific), ALEXA FLUORTM555 Donkey Anti‐mouse IgG (H + L) (2387458, Thermo Fisher Scientific).

### 2.5 Stereotaxic injection

The fur on the mouse's head was shaved using a hair trimmer to expose the desired injection sites. The exposed area revealed a distinct cross shape, with the intersection point representing the Bregma point. Subsequent brain region coordinates were determined relative to this point. The coordinates for the brain regions involved in this study were as follows: IC coordinates (AP: +0.2 mm; ML: −3.7 mm; DV: 4.1 mm) and PVT coordinates (AP: −1.45 mm; ML: −0.05 mm; DV: 3.1 mm). A homemade microsyringe was used to extract the virus for injection. The needle tip was positioned at the Bregma point, and then, it was moved to the target brain region according to the coordinates. The needle was lowered to the desired depth, and the virus was injected at a rate of 20 nL/min. After completing the viral injection, the needle was kept in place for 10 min before removal. Following the injection, the mice were placed under a heat lamp to maintain body temperature until they regained consciousness and returned to the animal facility.

### 2.6 In vivo fiber photometry

Fiber photometry is widely used to detect and record neuronal calcium activity based on fluorescent calcium indicator. GCaMP has an exceptionally high sensitivity to calcium ions and thus effectively indicates changes in calcium activity through instantaneous changes in fluorescence signal intensity.^25^, ^26^, ^27^ To assess the calcium ion activity of glutamatergic neurons in the IC, at the sixth week after birth, AAV2/9‐vglut2‐GCaMP6s (BrainVTA, Wuhan, China) virus was injected into the IC region of both NCI and CON group mice (150 nL per injection). Optical fibers (OD 200 mm, NA 0.37) were implanted into the IC brain region. After 3 weeks of viral expression, calcium imaging was performed during stimulation at 20, 40, 60, and 80 mmHg, respectively.

### 2.7 Abdominal electromyography (EMG) recording

The animals received abdominal EMG implantation 1 week prior to EMG recording. The fur on the abdomen and the neck of the mice was removed using an animal hair trimmer. The abdomen was then cut open using forceps and scissors. Electrode wires, approximately 1 cm in length, were implanted into the muscles on both sides of the abdomen. The wires were threaded subcutaneously from the abdomen to the back of the neck, brought out through the skin, and secured with adhesive film. The abdomen was sutured using a surgical needle and thread. Once the suturing was completed, the mice were placed under a heat lamp until they regained consciousness.

The distention protocol consists of four different pressures at 20, 40, 60 and 80 mmHg. Each pressure stimulus was applied for 20 s with a 2‐min interval between consecutive distensions to prevent tissue damage and to allow the mice to rest. During CRD distension, EMG activity was recorded under different pressure stimuli and subsequently used to assess the mice's colorectal visceral pain response. Finally, the Acknowledge software program was used to calculate the area under the curve (AUC) of the electromyographic signals over a 20 s period. All colorectal visceral pain behavioral tests were conducted under blinded conditions.

### 2.8 Optogenetics and chemogenetics

After viral AAV2/9‐hSyn‐DIO‐eNpHR3.0‐EYFP, AAV2/9‐hSyn‐DIO‐hChR2‐EYFP, AAV2/9‐vglut2‐hM3Dq‐mCherry, or AAV2/9‐vglut2‐hM4Di‐mCherry (BrainVTA, Wuhan, China) injection, mice were allowed a two‐week recovery period. Subsequently, abdominal electromyography (EMG) implantation surgery was performed. After an additional week of rest, optogenetic experiments were conducted using a 5 mW optogenetic system (Alpha Omega Engreth, Israel) for yellow light illumination (589 nm, continuous mode) and 3 mW blue light illumination (473 nm, 20 Hz, 5 ms pulse width). EMG signals from the abdominal muscles were recorded both during light stimulation and in the absence of light stimulation. A combined approach of chemogenetics and optogenetics was used to investigate the role of the PVT‐IC neural circuit in colorectal visceral pain. The method of CNO injection involved intraperitoneal injection at a concentration of 0.33 mg/mL with a total volume of 0.2 mL.

### 2.9 Data analysis and statistics

All data in this study are presented as mean ± standard error of the mean (SEM). Statistical analysis was conducted using Prism 6 (GraphPad) software. Normal distribution of data was analyzed using the Kolmogorov–Smirnov test. Differences in groups with normally distributed data were analyzed using the two‐sample *t*‐test, two‐way repeated‐measures analysis of variance (ANOVA); data that do not exhibit a normal distribution were analyzed using Mann–Whitney test. Statistical significance was considered when *p* < 0.05, indicating a significant difference.

## 3 RESULTS

### 3.1 NCI induced c‐Fos overexpression in the glutamatergic neurons of IC brain region

**FIGURE 1:**
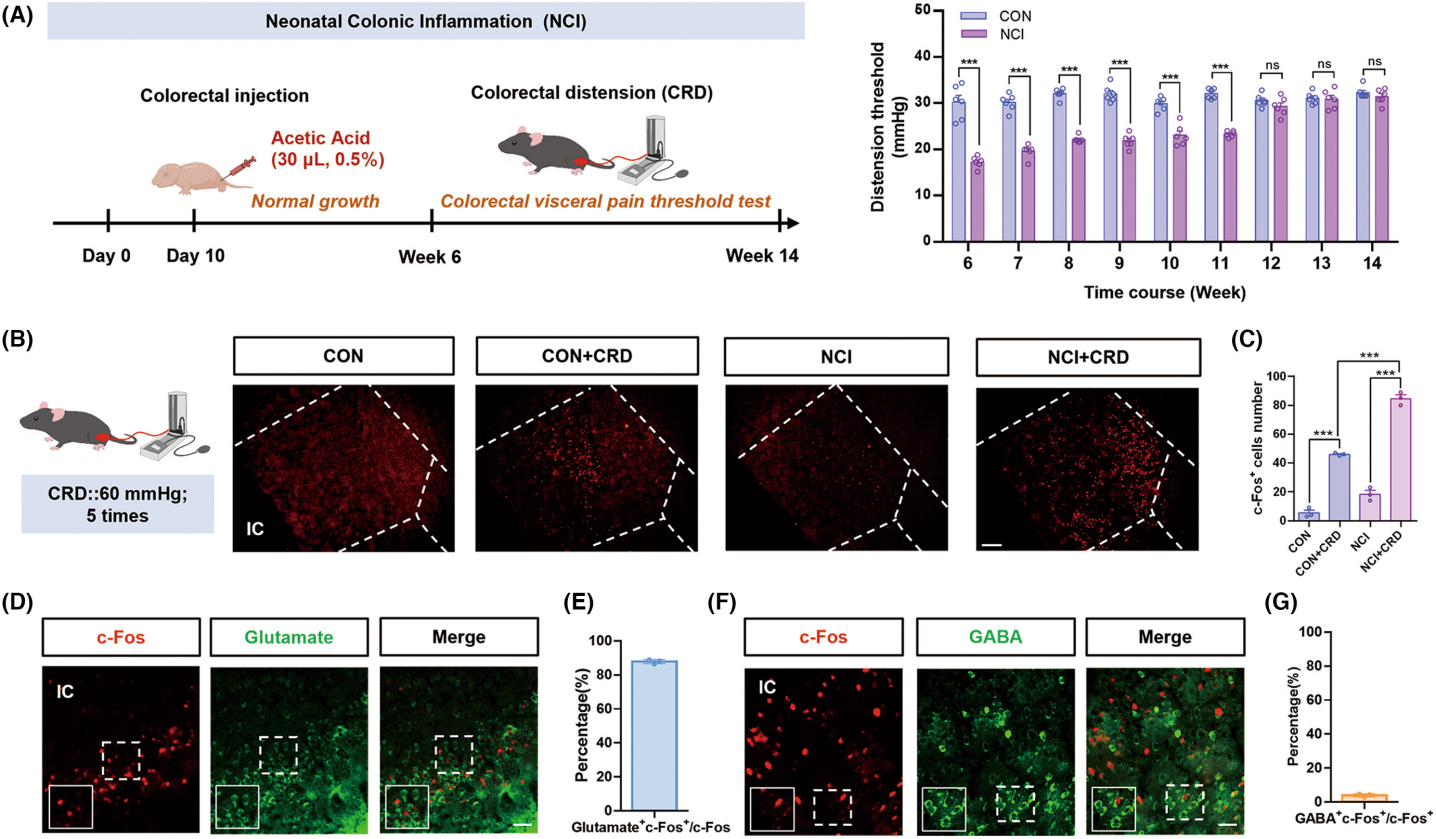
NCI induced higher c‐Fos expression in glutamatergic neurons in the IC brain region in response to CRD. (A) NCI model flowchart and the statistical graph of visceral pain threshold in CON mice and NCI mice from the age of week 6 to week 14 (***p < 0.001, two‐way ANOVA followed by Sidak's multiple comparison test, *n* = 6 for each group). (B) Representative c‐Fos immunofluorescence in the IC region (Scale: 20 μm). (C) Statistical graph of c‐Fos positive cells in the IC region of CON, CON + CRD, NCI, and NCI + CRD groups of mice (^#^ *p* < 0.05; ***p < 0.001, one‐way ANOVA followed by Tukey's multiple comparisons test, *n* = 3 brain sections from 3 mice). (D) Representative immunofluorescence images: from left to right, c‐Fos, glutamate, and their merge (Scale: 50 μm). (E) Percent of c‐Fos and glutamate colabeled neurons in total c‐Fos positive neurons (*n* = 3 brain sections from 3 mice). (F) Representative immunofluorescence images: from left to right, c‐Fos, GABA, and their merge (Scale: 50 μm). (G) Percent of c‐Fos and GABA colabeled neurons in total c‐Fos positive neurons (*n* = 3 brain sections from 3 mice). CRD, colorectal distension; IC, insular cortex; NCI, neonatal colonic inflammation.

Previous studies in our laboratory have shown that colonic injection of acetic acid induces colorectal visceral pain in adult SD rats,^28^ known as the Neonatal Colonic Inflammation (NCI) model. This model mimics the clinical manifestations of patients with irritable bowel syndrome (IBS) and provides convenience for the basic research of IBS. Based on this, we adjusted the injection volume of acetic acid and found that colonic injection of 30 μL of 0.5% acetic acid in male neonatal mice (10 days old) established a stable NCI mouse model. When the mice reached 6 weeks of age, compared to the CON group mice, the NCI group mice showed a significant decrease in colorectal visceral pain threshold and persisted until the 11th week (Figure 1A). CRD induced a significant increase in c‐Fos expression in the IC region of both CON group and NCI group. Furthermore, c‐Fos expression in the NCI + CRD group was significantly higher than the CON + CRD group (Figure 1B,C). A large number of c‐Fos‐positive cells co‐localized with the marker for glutamatergic neurons (Figure 1D,E), while a smaller number co‐localized with the marker for GABAergic neurons (Figure 1F,G). These findings suggest that glutamatergic neurons in the IC brain region may play an important role in mediating colorectal visceral pain induced by NCI.

### 3.2 NCI enhanced calcium activity of glutamatergic neurons in the IC in response to CRD

**FIGURE 2:**
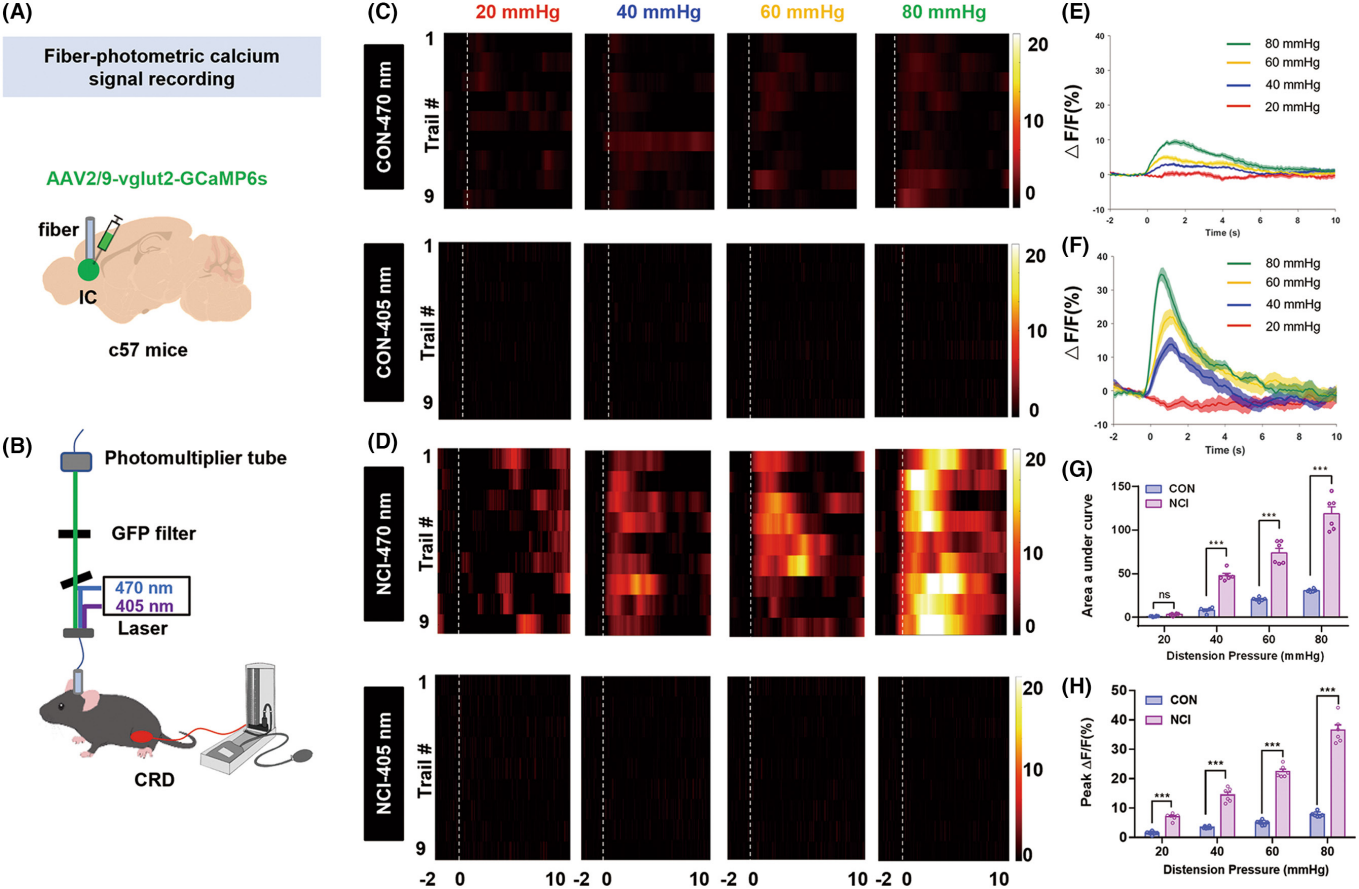
NCI significantly enhanced the IC brain region glutamatergic neurons calcium activity induced by CRD. (A) A diagram illustrating viral injection. (B) Optical recording of calcium ion activity in mice during CRD stimulation. (C, D) Heatmap of IC calcium activity of CON and NCI mice in response to 20, 40, 60, and 80 mmHg stimulation, respectively. (E, F) Pattern diagrams of calcium ion transient changes in CON and NCI mice in response to CRD stimulation. (G) Area under the curve of calcium ion activity in CON and NCI mice in response to CRD stimulation (***p < 0.001, two‐way ANOVA followed by Sidak's multiple comparison test, *n* = 6 mice for each group). (H) Average peak Δ*F*/*F* of calcium ion activity in glutamatergic neurons of the IC region in CON and NCI mice (**p < 0.01; ***p < 0.001, two‐way ANOVA followed by Sidak's multiple comparison test, *n* = 6 mice for each group). CRD, colorectal distension; IC, insular cortex.

To further elucidate the functional relationship between colorectal visceral pain in NCI mice and the activation of glutamatergic neurons in the IC region, we employed in vivo fiber photometry recording technology to monitor real‐time calcium activity of glutamatergic neurons in the IC of mice injected with the AAV2/9‐vglut2‐GCaMP6s virus (Figure 2A,B). In both CON and NCI group mice, as the intensity of CRD stimulation increased, calcium activity was significantly enhanced (Figure 2C–F). Statistical analysis of the AUC of glutamatergic neuronal calcium activity in the IC region between the CON and NCI groups revealed no significant difference at 20 mmHg stimulation. However, calcium responses at 40, 60, and 80 mmHg were significantly increased in the NCI mice (Figure 2G, ***p < 0.001, two‐way ANOVA followed by Sidak's multiple comparison test, *n* = 6 for each group). Consistently, peak values of glutamatergic neuronal calcium activity in the IC region between was significantly enhanced at 20, 40, 60, and 80 mmHg (Figure 2H, **p < 0.01, ***p < 0.001, two‐way ANOVA followed by Sidak's multiple comparison test, *n* = 6 for each group). These findings further suggest the potential involvement of glutamatergic neurons in the IC region in the development of chronic colorectal visceral pain in NCI mice.

### 3.3 Optogenetic manipulation of IC glutamatergic neuronal activity affected colorectal visceral pain responses

**FIGURE 3:**
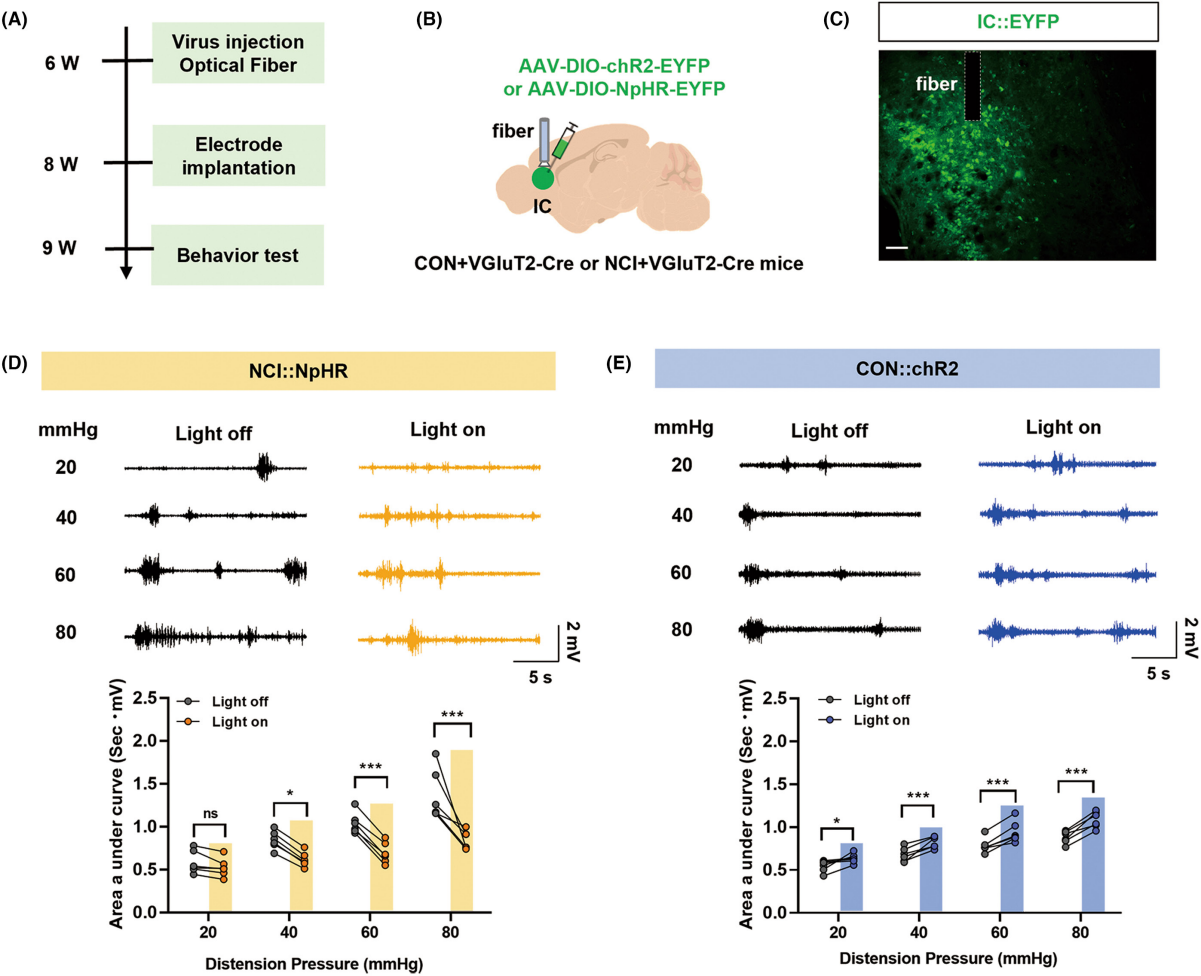
Optogenetic manipulation of glutamatergic neurons in the IC region altered colorectal visceral pain responses to CRD. (A) Optogenetic manipulation of glutamatergic neurons in the IC region's experimental flowchart. (B) A diagram illustrating viral injection and ceramic implantation in the IC region of NCI + VGluT2‐Cre mice. (C) Fluorescent representation of viral expression in the IC (Scale: 20 μm). (D) Representative electromyography (EMG) responses to CRD of NCI + VGluT2‐Cre mice under normal conditions (black) or upon yellow light stimulation (yellow) and statistical graph of EMG AUC (*p < 0.05; **p < 0.01; ***p < 0.001, two‐way ANOVA followed by Sidak's multiple comparison test, *n* = 6 mice for each group). (E) Representative EMG recordings of CON + VGluT2‐Cre mice under normal conditions (black) or during blue light stimulation (blue) and statistical graph of EMG AUC (*p < 0.05; **p < 0.01, two‐way ANOVA followed by Sidak's multiple comparison test, *n* = 6 mice for each group). AUC, area under the curve; CRD, colorectal distension; IC, insular cortex.

To specifically manipulate IC glutamatergic neuronal activity, NCI model was established in VGluT2‐Cre mice with injection of AAV2/9‐hSyn‐DIO‐NpHR‐EYFP or AAV2/9‐hSyn‐DIO‐hChR2‐EYFP viruses (200 nL) into the IC brain region. The DIO element carried by these viruses allows specific labeling of glutamatergic neurons in VGluT2‐Cre mice. Yellow light‐induced inhibition of glutamatergic neuronal activity in the IC brain region of NCI mice significantly reduced the colorectal visceral pain responses (AUC of EMG) (Figure 3D, *p < 0.05, **p < 0.01, ***p < 0.001, two‐way ANOVA followed by Sidak's multiple comparison test, *n* = 6 for each group). On the other hand, blue light‐induced activation of glutamatergic neurons in the IC brain region of CON mice significantly increased colorectal visceral pain responses in CON + VGluT2‐Cre mice, indicating the induction of colorectal visceral pain (Figure 3E, *p < 0.05, **p < 0.01, two‐way ANOVA followed by Sidak's multiple comparison test, *n* = 6 for each group). These results suggest that selectively altering the activity of glutamatergic neurons in the IC brain region may influence colorectal visceral pain.

### 3.4 Glutamatergic neurons in the PVT projected to glutamatergic neurons in the IC

**FIGURE 4:**
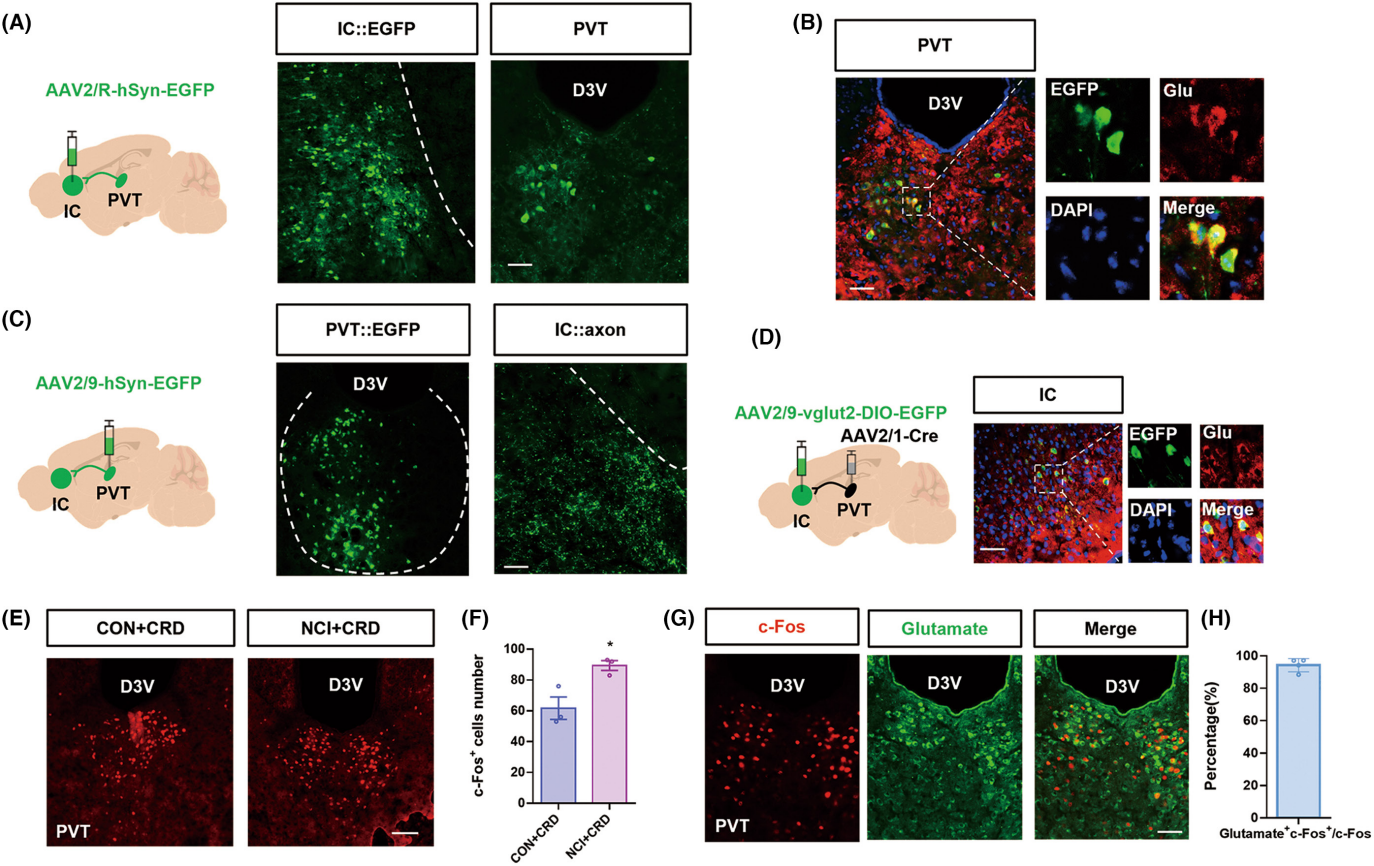
Identification of PVT‐IC neural pathway. (A) A diagram illustrating retrograde tracing virus injection into the IC. The virus fluorescence images obtained after virus injection in the IC brain region and PVT brain region clearly demonstrate cellular morphology (Scale: 50 μm). (B) Representative immunofluorescence images of EGFP, Glutamate, DAPI merged image in PVT (Scale: 50 μm). And a partial enlargement of the picture in the box eventually. (C) The virus fluorescence images obtained after virus injection in the PVT brain region clearly demonstrate cellular morphology, while the virus fluorescence images obtained in the IC brain region clearly demonstrate terminal morphology. (Scale: 50 μm). (D) A diagram illustrating viral injection into the IC and PVT, where the combination of these two viruses resulted in green fluorescence. Representative immunofluorescence images of EGFP, Glutamate, DAPI merged image in IC (Scale: 50 μm). And a partial enlargement of the picture in the box eventually too. (E) Representative c‐Fos immunofluorescence in the PVT upon CRD (Scale: 50 μm). (F) Quantification of c‐Fos positive cells in the PVT brain region of the CON + CRD and NCI + CRD groups (**p < 0.01, Mann–Whitney test, *n* = 3 brain sections from 3 mice). (G) Representative immunofluorescence results, from left to right, showing c‐Fos, glutamate, and the merged image (Scale: 50 μm). (H) Percentage of c‐Fos and glutamate colabeled neurons in c‐Fos positive cells (*n* = 3 brain sections from 3 mice). IC, insular cortex; PVT, paraventricular thalamus.

There is literature evidence confirming the projection from PVT to IC,^29^ but the specific neuronal projection types are unclear. We used viral tracing techniques and injected retrograde tracing virus AAV2/R‐hSyn‐EGFP (150 nL) into the IC brain region. Three weeks after virus injection, we observed virus‐infected neurons in the IC, while in the PVT, we clearly observed illuminated cell bodies indicating viral labeling (Figure 4A). To investigate the neuronal types projecting between these two nuclei, we combined viral tracing with immunofluorescence techniques. We found colocalization of the glutamatergic neurons with the PVT virus‐traced cell bodies (Figure 4B). To further confirm the existence of a circuit between the two regions, we injected anterograde tracing virus AAV2/9‐hSyn‐EGFP (150 nL) into the PVT and observed marked viral labeling in the IC, specifically in the axonal terminals (Figure 4C). Subsequently, viral tracing with AAV2/1‐Cre and AAV2/9‐vglut2‐DIO‐EGFP labeled cell bodies in the IC, which were also colabeled with glutamate (Figure 4D). These findings indicate that glutamatergic neurons in the PVT region project to glutamatergic neurons in the IC region.

The above results indicate the involvement of the IC in the regulation of colorectal visceral pain in NCI mice and that the PVT neurons project to the IC. The literature suggests that the PVT is involved in pain processing; thus, we hypothesized that the PVT may be involved in the regulation of colorectal visceral pain in NCI mice. To test this hypothesis, we employed immunofluorescence techniques to investigate the activity of the PVT under NCI induction. Immunofluorescence results demonstrated that compared to the CON group, CRD stimulation in the NCI mice significantly increased the number of c‐Fos‐positive cells in the PVT region (Figure 4E,F, *p < 0.05, Mann–Whitney test, *n* = 3 for each group). To further determine the neuronal types involved in the PVT region, we performed co‐staining of c‐Fos and glutamate using immunofluorescence techniques and observed a substantial overlap between the two markers (Figure 4G,H). This suggests the glutamatergic neurons in the PVT region might be involved in colorectal visceral pain of NCI mice.

### 3.5 Optogenetic modulation of glutamatergic neuronal activity in the PVT region affected colorectal visceral pain response

**FIGURE 5:**
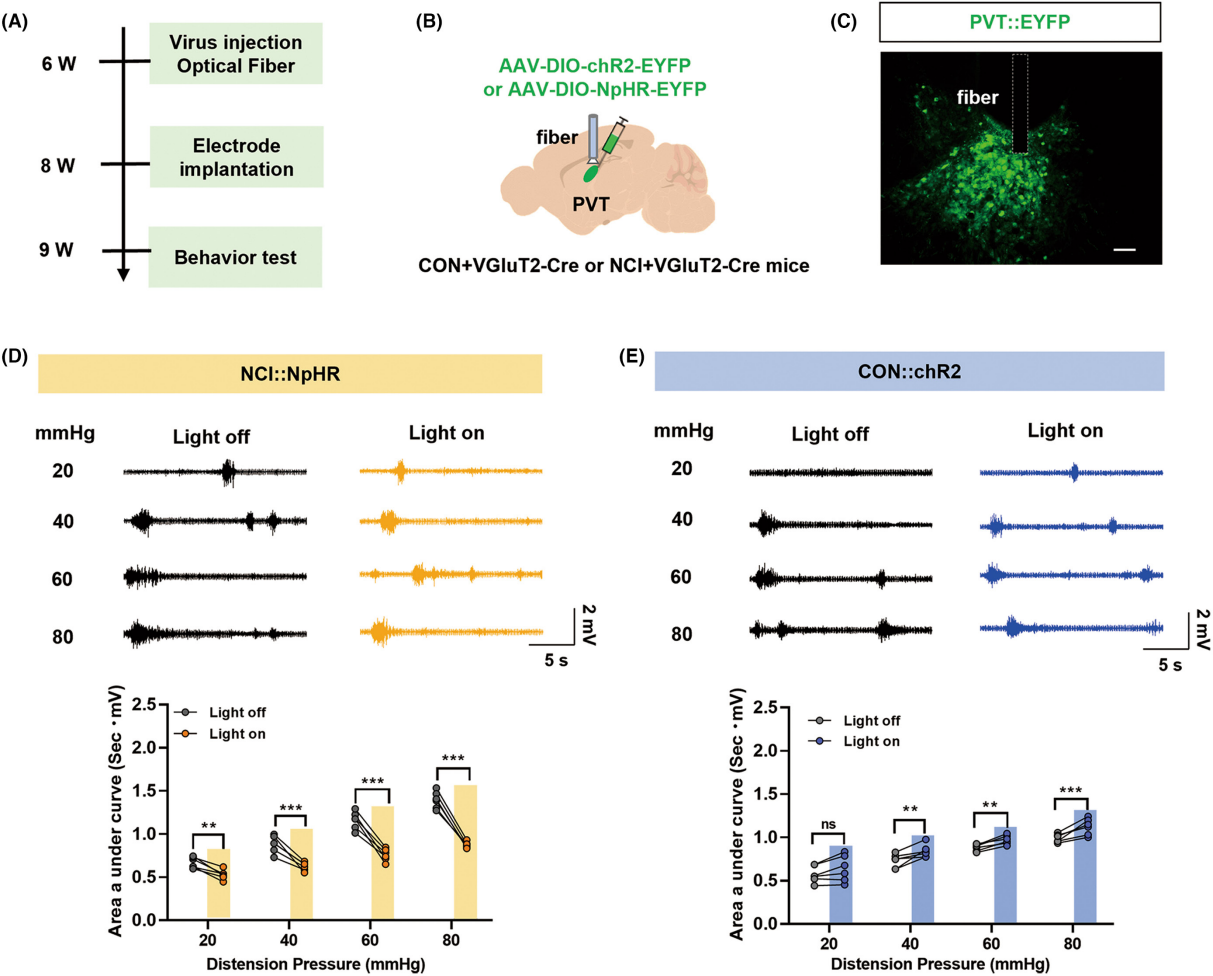
Optogenetic manipulation of glutamatergic neurons in the PVT region altered colorectal visceral pain responses to CRD. (A) Optogenetic manipulation of glutamatergic neurons in the PVT region's experimental flowchart. (B) Diagram illustrating the injection of virus and ceramic implantation in the PVT brain region of NCI + VGluT2‐Cre mice. (C) Fluorescence diagram showing virus expression in the PVT (Scale: 20 μm). (D) EMG responses to CRD of NCI + VGluT2‐Cre mice under normal conditions (black) or upon blue light stimulation (blue) and statistical graph of EMG AUC (**p < 0.01 and ***p < 0.001, two‐way ANOVA followed by Sidak's multiple comparison test, *n* = 6 mice for each group). (E) Representative EMG recordings of CON + VGluT2‐Cre mice under normal conditions (black) or during blue light stimulation (blue) and statistical graph of EMG AUC (*p < 0.05 and **p < 0.01, two‐way ANOVA followed by Sidak's multiple comparison test, *n* = 6 mice for each group). AUC, area under the curve; CRD, colorectal distension; EMG, electromyography; PVT, paraventricular thalamus.

To further validate the functional involvement of PVT glutamatergic neurons in colorectal visceral pain of NCI mice, we employed a combination of abdominal electromyography (EMG) and optogenetics. In the PVT region of VGluT2‐Cre mice, we injected AAV2/9‐hSyn‐DIO‐NpHR‐EYFP or AAV2/9‐hSyn‐DIO‐hChR2‐EYFP viruses at a volume of 200 nL (Figure 5A–C). The results revealed that selective inhibition of glutamatergic neurons in the NCI group significantly reduced the EMG AUC (Figure 5D, **p < 0.01, ***p < 0.001, two‐way ANOVA followed by Sidak's multiple comparison test, *n* = 6 for each group). Furthermore, specific activation of the PVT glutamatergic neurons of the CON+VGluT2‐Cre mice using blue light significantly increased the AUC, indicating development of colorectal visceral pain (Figure 5E, *p < 0.05, **p < 0.01, two‐way ANOVA followed by Sidak's multiple comparison test, *n* = 6 for each group). These findings suggest that selectively modulating the activity of glutamatergic neurons in the PVT region alters colorectal visceral pain, and thus, PVT glutamatergic neurons contribute to the development of colorectal visceral pain in NCI mice.

### 3.6 PVT‐IC neural pathway regulated visceral pain in NCI mice

**FIGURE 6:**
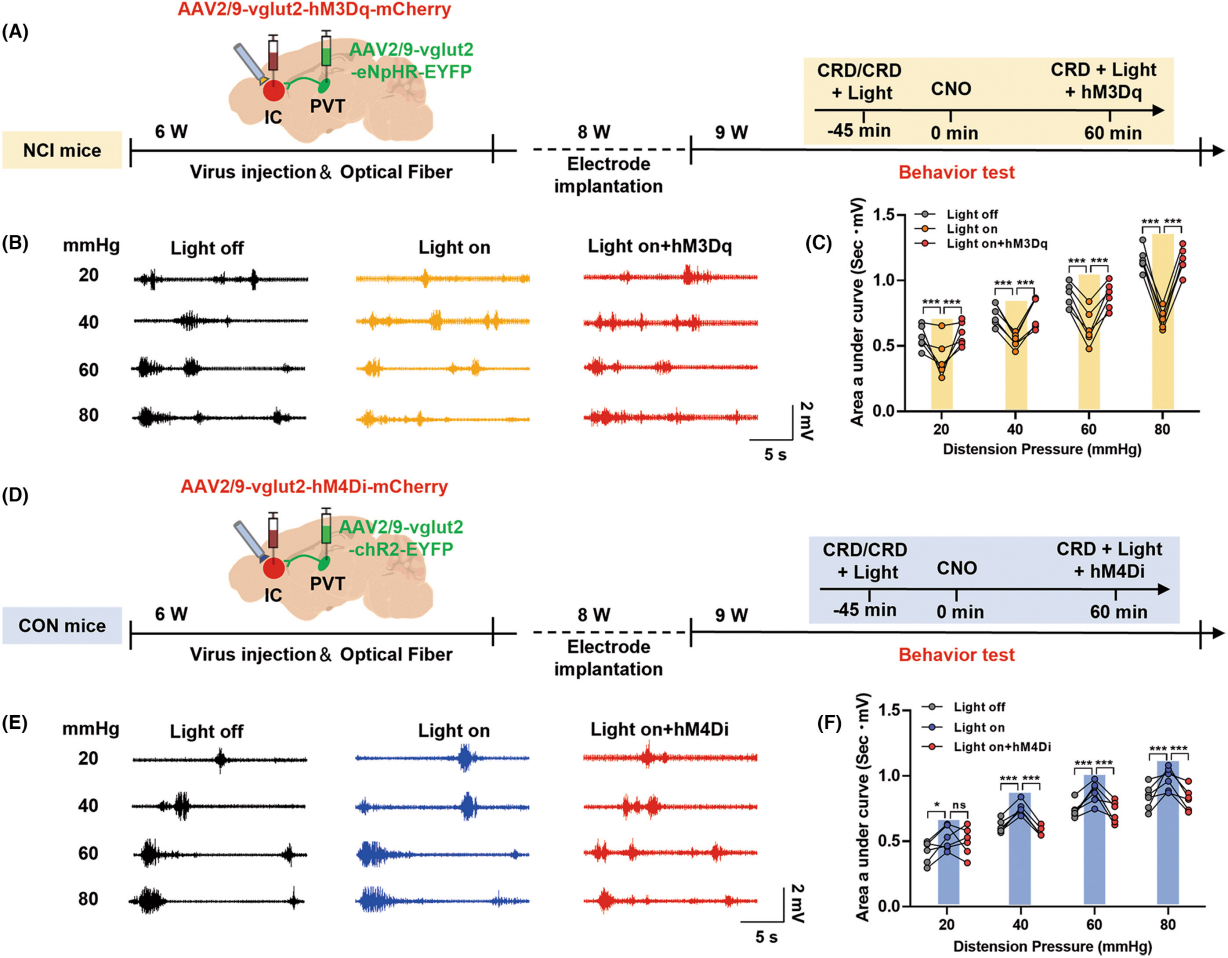
Inhibiting the PVT‐IC pathway alleviated colorectal visceral pain in mice, while activating this pathway promoted colorectal visceral pain. (A) Inhibiting the PVT‐IC pathway experimental design and experimental flowchart in NCI mice. (B) Diagram illustrating electromyography (EMG) recordings. Black represents EMG recordings of NCI mice without light stimulation under 20, 40, 60, and 80 mmHg stimulation. Yellow represents EMG recordings with yellow light stimulation under 20, 40, 60, and 80 mmHg stimulation. Red represents EMG recordings with yellow light and chemogenetic stimulation under 20, 40, 60, and 80 mmHg stimulation. (C) Statistical analysis of the EMG recordings from diagram B using the area under the curve (AUC) analysis with significance levels of *p < 0.05; **p < 0.01; and ***p < 0.001 (two‐way ANOVA followed by Sidak's multiple comparison test, *n* = 6 mice for each group). (D) Activating the PVT‐IC pathway experimental design and experimental flowchart in CON mice. (E) Diagram illustrating EMG recordings. Black represents EMG recordings of CON mice without light stimulation under 20, 40, 60, and 80 mmHg stimulation. Blue represents EMG recordings with blue light stimulation under 20, 40, 60, and 80 mmHg stimulation. Red represents EMG recordings with blue light and chemogenetic stimulation under 20, 40, 60, and 80 mmHg stimulation. (F) Statistical analysis of the EMG recordings from diagram C using the AUC analysis with significance levels of *p < 0.05; **p < 0.01; and ***p < 0.001 (two‐way ANOVA followed by Sidak's multiple comparison test, *n* = 6 mice for each group). NCI, neonatal colonic inflammation.

The above experiments demonstrated that glutamatergic neurons in both the PVT and IC regions were involved in the development of colorectal visceral pain in NCI mice. Moreover, morphological evidence showed direct projections from glutamatergic neurons in the PVT to glutamatergic neurons in the IC. Based on these findings, we hypothesized that glutamatergic neurons in the PVT mediate colorectal visceral pain in mice by modulating the activity of glutamatergic neurons in the IC. To test this hypothesis, we employed a combination of optogenetics and chemogenetics. First, we injected optogenetic viruses AAV2/9‐vglut2‐eNpHR‐EYFP into the PVT region and chemogenetic viruses AAV2/9‐vglut2‐hM3Dq‐mCherry into the IC region (Figure 6A). The results showed that optogenetic inhibition of PVT glutamatergic neuronal terminals in the IC using yellow light significantly alleviated colorectal visceral pain responses in NCI mice; conversely, activation of IC glutamatergic neurons by intraperitoneal injection of CNO (a chemical activator) reversed the analgesic effect of yellow light (Figure 6B,C, *p < 0.05, **p < 0.01, ***p < 0.001, two‐way ANOVA followed by Sidak's multiple comparison test, *n* = 6 for each group). Then, we injected optogenetic viruses AAV2/9‐vglut2‐chR2‐EYFP into the PVT region and chemogenetic viruses AAV2/9‐vglut2‐hM4Di‐mCherry into the IC region (Figure 6D). Optogenetic activation of PVT glutamatergic neuron terminals in the IC using blue light induced colorectal visceral pain in control mice, while chemogenetic inhibition of IC glutamatergic neurons reversed the sensitizing effect of blue light (Figure 6E,F, *p < 0.05, **p < 0.01, ***p < 0.001, two‐way ANOVA followed by Sidak's multiple comparison test, *n* = 6 for each group). These results indicate that neural signals transmitted from PVT glutamatergic neurons to IC glutamatergic neurons contribute to colorectal visceral pain in NCI mice.

## 4 DISCUSSION

Emerging evidence indicates the involvement of central mechanisms in chronic colorectal visceral pain, but the neural circuit mechanisms remain poorly understood. This study demonstrated direct projections from the PVT glutamatergic neurons to the IC glutamatergic neurons, and selective manipulation of this neural circuit modulated colorectal visceral pain in NCI mice. These findings provide a novel insight into central mechanism of colorectal visceral pain and potential therapeutic targets for IBS.

As part of the brain limbic system, the IC plays a vital role in the transmission and encoding of sensory information in the body, maintaining normal physiological activity.^30^, ^31^, ^32^ Previous research has indicated that abnormal activation of IC neurons mediates pain regulation and emotional changes in mice, primarily through the involvement of glutamatergic neurons.^33^ The current study found that NCI significantly increased the c‐Fos expression and calcium activity in response to CRD in the IC region, predominantly glutamatergic neurons. Furthermore, optogenetic inhibition of glutamatergic neurons in the IC alleviated visceral pain in NCI mice, while optogenetic activation of glutamatergic neurons in the IC induced colorectal visceral pain in normal mice. These results suggest that IC glutamatergic neurons play an important role in colorectal visceral pain of IBS.

Our study identified a PVT‐IC pathway that is critical in mediating the development of colorectal visceral pain behavior. By employing multiple viral tracing strategies, we demonstrated direct projections from the PVT region to the IC region. As an important region in the thalamus, PVT serves as a crucial relay station for peripheral signals.^34^ Existing research has shown that the PVT region is involved in the regulation of various behaviors, such as fear, sleep–wake cycles, depressive, and addiction.^35^, ^36^, ^37^, ^38^ The PVT region predominantly consists of glutamatergic neurons,^16^ yet it projects to multiple nuclei to regulate different behavioral responses.^39^, ^40^ This study utilized immunofluorescence techniques and optogenetics to demonstrate, both morphologically and functionally, the involvement of PVT glutamatergic neurons in the development of colorectal visceral pain in NCI mice. Diverse functions of PVT may be mediated by different receptors; thus, further research should investigate molecular mechanisms underlying the contribution of PVT in colorectal visceral pain.

The glutamatergic neurons regulate different behaviors by forming neural circuits with other types of neurons. For example, neural circuits formed by glutamatergic neurons with other glutamatergic neurons modulate sleep–wake cycles, cognitive impairments, and learning and memory deficits.^41^, ^42^, ^43^ In addition, our research has previously shown that other brain circuits glutamatergic neurons form neural circuits with glutamatergic neurons that modulate visceral pain.^27^, ^44^ In this study, viral tracing results revealed that excitatory neurons in the PVT projected to the IC excitatory neurons. Immunofluorescence analysis indicated that these excitatory neurons were primarily glutamatergic neurons. Further optogenetic experiments demonstrated that both the PVT and IC brain region, specifically the glutamatergic neurons, were involved in mediating colorectal visceral pain in NCI mice. Therefore, we examined if the terminals of PVT glutamatergic neurons regulated the activity of IC glutamatergic neurons to modulate the development of colorectal visceral pain in NCI mice. By employing a combination of optogenetics, chemogenetics, and electromyography recording, we revealed that manipulating the terminals of PVT neurons in the IC using optogenetic techniques altered colorectal visceral pain in mice, which were reversed by chemogenetic modulation of the neural activity in the IC. These findings suggest that PVT glutamatergic neurons mediate chronic visceral pain by regulating the activity of IC glutamatergic neurons. Future studies should confirm the specificity of this neural circuit in multiple different pain models to find the most promising approaches for the treatment of chronic visceral pain.

In summary, the present study revealed a critical role of the glutamatergic neuronal connection between the PVT‐IC in colorectal visceral pain. Our work will strengthen fundamental research on the involvement of central nervous system in colorectal visceral pain and provide potential therapeutic targets towards effective treatment.

## AUTHOR CONTRIBUTIONS

Fu‐Chao Zhang performed experiments, analyzed data, and prepared the manuscript. Ying‐Xue Wei and Rui‐Xia Weng performed experiments, analyzed data, and prepared the figures. Qi‐Ya Xu and Y.Y performed experiments and analyzed data. Rui Li analyzed data and prepared the manuscript. Guang‐Yin Xu designed experiments, supervised the experiments, and finalized the manuscript. All the authors have read and approved the paper.

## CONFLICT OF INTEREST STATEMENT

No conflicts of interest, financial or otherwise, are declared by the authors.

## Data Availability

The data that support the findings of this study are available from the corresponding author upon reasonable request.
